# A Tale of Two Fimbriae: How Invasion of Dendritic Cells by *Porphyromonas gingivalis* Disrupts DC Maturation and Depolarizes the T-Cell-Mediated Immune Response

**DOI:** 10.3390/pathogens11030328

**Published:** 2022-03-08

**Authors:** Mohamed M. Meghil, Mira Ghaly, Christopher W. Cutler

**Affiliations:** Department of Periodontics, The Dental College of Georgia at Augusta University, Augusta, GA 30912, USA; mmeghil@augusta.edu (M.M.M.); mghaly@augusta.edu (M.G.)

**Keywords:** dendritic cells, T-cells, immunology, *Porphyromonas gingivalis*, periodontitis

## Abstract

*Porphyromonas gingivalis* (*P. gingivalis*) is a unique pathogen implicated in severe forms of periodontitis (PD), a disease that affects around 50% of the US population. *P. gingivalis* is equipped with a plethora of virulence factors that it uses to exploit its environment and survive. These include distinct fimbrial adhesins that enable it to bind to other microbes, colonize inflamed tissues, acquire nutrients, and invade cells of the stroma and immune system. Most notable for this review is its ability to invade dendritic cells (DCs), which bridge the innate and adaptive immune systems. This invasion process is tightly linked to the bridging functions of resultant DCs, in that it can disable (or stimulate) the maturation function of DCs and cytokines that are secreted. Maturation molecules (e.g., MHCII, CD80/CD86, CD40) and inflammatory cytokines (e.g., IL-1b, TNFa, IL-6) are essential signals for antigen presentation and for proliferation of effector T-cells such as Th17 cells. In this regard, the ability of *P. gingivalis* to coordinately regulate its expression of major (fimA) and minor (mfa-1) fimbriae under different environmental influences becomes highly relevant. This review will, therefore, focus on the immunoregulatory role of *P. gingivalis* fimbriae in the invasion of DCs, intracellular signaling, and functional outcomes such as alveolar bone loss and immune senescence.

## 1. Introduction

### 1.1. Periodontitis

Periodontitis (PD) is a chronic inflammatory disease resulting from a microbial dysbiosis. Unresolved inflammation in the soft and hard tissues surrounding the teeth results in the loss of supporting tissue and eventually loss of the teeth. While bacterial biofilm is a causative factor, smoking and diabetes remain the strongest risk factors for the severity and progression of periodontal disease in a susceptible host. Periodontitis affects almost half of the US population and, as the population ages, the prevalence of periodontal disease will also increase. Eke et al. reported on the prevalence of periodontitis from the NHANES (2009–2010 and 2011–2012). In 2009 to 2012, 46% of US adults, representing 64.7 million people, had PD, with 8.9% having severe PD. The prevalence of PD was higher among males and was positively associated with increasing age through as yet unclear mechanisms, though immune senescence has been proposed [1,2]. PD prevalence was highest in Hispanics (63.5%) and non-Hispanic blacks (59.1%), followed by non-Hispanic Asian Americans (50.0%), and lowest in non-Hispanic whites (40.8%). Prevalence varied two-fold between the lowest and highest levels of socioeconomic status, whether defined by poverty or education [3]. Not only has smoking been shown to affect the humoral and cellular immune responses but also the vasculature, cell signaling mechanisms, tissue homeostasis, and even the composition and quantity of the subgingival microflora [4,5,6,7,8,9,10,11]. Data derived from the NHANES III study suggested that up to 42% of PD cases in the US can be attributed to current smoking and 11% to former smoking. In longitudinal studies, adjusted for co-variates, smoking has been found to confer a statistically significant increased risk for PD progression [12,13,14,15,16,17,18]. Smoking also has a negative effect on the outcome of both non-surgical and surgical periodontal therapy: Current smokers exhibit poorer responses than former or never smokers [19,20,21,22,23,24,25,26,27,28,29,30]. Smoking cessation results in beneficial effects on periodontal status, and former smokers have the potential to experience periodontal stability similar to non-smokers [31,32,33,34,35]. The effects of diabetes mellitus (DM) span multiple organs throughout the entire body. The oral cavity and, more precisely, the periodontal apparatus are also affected [36,37,38,39]. Chávarry et al. confirmed a strong association between type 2 DM and PD, but concluded that the evidence for type 1 DM was weaker in a recent meta-analysis [40]. Poor metabolic control and extended duration of DM increase the adverse effects on a patient’s periodontal status [41,42,43,44]. Furthermore, previous studies have reported the relationship between poor metabolic control and the severity as well as the progression of PD [45,46,47,48,49,50]. Similarly to smoking, patients with poorly controlled DM display a poorer treatment outcome of periodontal treatment [51], whereas well-controlled diabetic patients and non-diabetic subjects have similar responses [52,53,54]. The interaction between DM and PD seems to be a “two-way street”: Just as an increased severity of periodontal tissue destruction is observed in subjects with DM, studies indicate a higher incidence of DM complications and poorer metabolic control of diabetes in PD patients [50]. Therefore, it is of importance that the diabetic control be established to increase control of the periodontal condition as well as the periodontal condition be addressed in conjunction with medications, diet, and exercise to aid in maintaining diabetic control. The consensus report of the 1996 World Workshop in Periodontics identified three species, Actinobacillus actinomycetemcomitans (now Aggregatibacter actinomycetemcomitans) [55], *P. gingivalis*, and Bacteroides forsythus (now Tannerella forsythia) [56,57], as causative factors for PD. As such, much research has been devoted to these three species but they should not be considered to be the only causative pathogens because only approximately 50% of the bacteria of the oral cavity are currently recognized [58]. Two decades of research have contributed to our understanding of the role of specific periodontal bacteria as risk factors for PD and have clarified that (1) the clinical presentation of a patient is determined by the burden of the exposure to the specific micro-organisms rather than its mere presence, (2) within a microbial species, there may be clonal types that are more virulent and can cause more or less disease at a faster or slower rate. and (3) reducing said pathogens to undetectable levels from the subgingival microbiome results in improved periodontal clinical markers [13,14,59,60,61,62,63,64,65,66,67]. Based on systematic reviews, it is clear that an antimicrobial approach consisting of the removal of subgingival plaque with or without adjunctive local or systemic antibiotics followed by adequate maintenance care is currently the single most successful and consistent strategy in the treatment of PD and maintenance of long-term stability [68,69,70]. 

### 1.2. P. gingivalis Fimbriae and the Prevalence of Periodontal Disease

*P. gingivalis* is a Gram-negative, black-pigmented, anaerobic coccobacillus. *P. gingivalis* is considered a keystone pathogen in the development of PD [71]. In addition, it is part of the anaerobic bacterial complex, known as the red complex, consisting of *Treponema denticola* (*T. denticola*) and *Tannerella forsythia* (*T. forsythia*), which has been implicated in severe forms of PD [72]. The progression from gingivitis to PD is associated with a dramatic shift from a symbiotic, aerobic microbial community bacteria to a dysbiotic, anaerobic polymicrobial complex that elicits a pro-inflammatory immune response [73]. *P. gingivalis* is equipped with an arsenal of virulence factors, including fimbriae, cysteine proteinases, hemagglutinins, and LPS, which together strongly support its pathogenicity. This occurs though a combination of binding to host cells and to other microbes, using blood hemin for growth and cellular invasion. This review focused on the role of *P. gingivalis* fimbria. 

Fimbriae are appendages present on the outer surface that are involved in the *P. gingivalis* cell membrane and greatly contribute to its virulence [74]. *P. gingivalis* fimbriae play a crucial role in nearly all interactions between the bacterium and the host, as well as with other bacteria. More importantly, *P. gingivalis* fimbriae have been identified as a key factor in its adhesion, invasion, and colonization of the oral mucosa [75,76]. *P. gingivalis* has two distinct types of fimbriae: long and short fimbriae [77,78]. The long fimbriae (FimA) are also known as major fimbriae while the short fimbriae (Mfa1) are known as minor fimbriae. Both *P. gingivalis* fimbriae are involved in the initial attachment and organization of the biofilm and the attachment to other bacteria [79]. Most notably, the fimbriae of *P. gingivalis* are required for invasion of DCs. The minor fimbriae, comprised of a 67-kDa glycoprotein that is encoded by the *mfa1* gene [80], targets the C-type lectin DC-SIGN on DCs for entry [78] and survival within [81]. The major fimbriae are composed of a 41-kDa protein called fimbrillin and encoded by the *fimA* gene [82]. The *fimA* gene has been classified into six types (I, Ib, II, III, IV, V) [83,84,85], based on nucleotide sequence variation. The difference between fimA genotypes in the context of pathogenicity and ability to adhere and invade human cells has been studied before. Studies using recombinant FimA protein corresponding to fimA genotype II have reported that genotype II has a greater ability to adhere to and invade human epithelial cells than FimA corresponding to the protein from other genotypes. Furthermore, it was shown that *fimA* genotypes II, Ib, and IV cause stronger infectious symptoms and inflammatory response in animal models, relative to *fimA* genotypes I and III [86,87,88]. 

Results of various clinical studies also support findings that nucleotide variation of the *fimA* gene is contributing to the virulence of *P. gingivalis* strains. Isolates with *fimA* genotypes II, IV, and Ib have been shown to be significantly more prevalent than isolates with other genotypes [84,85,89,90,91,92,93,94]. More recently, a meta-analysis by Wang et al. showed that the *fimA* II and *fimA* IV genotypes of *P. gingivalis* are highly prevalent in patients with PD [95], suggesting that these two genotypes may be related to the pathogenesis and progression of PD.

### 1.3. Regulation of P. gingivalis Fimbriae

**Figure 1:**
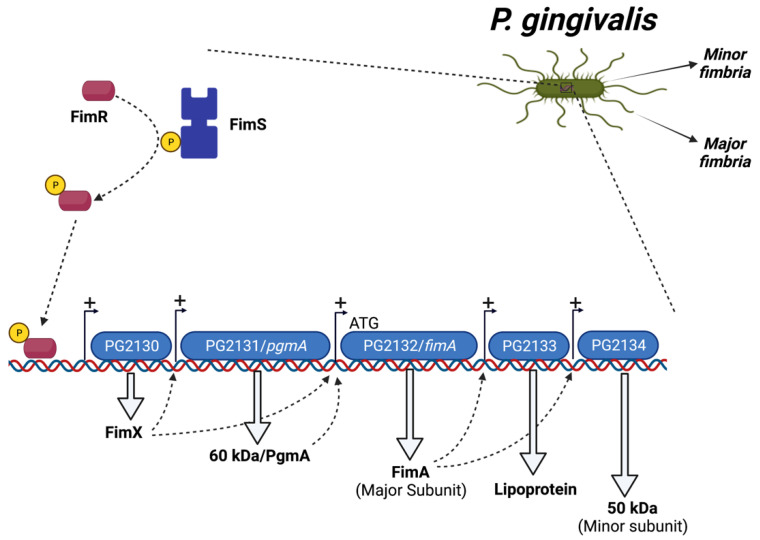
Regulation of *P. gingivalis* FimA fimbria by a FimS-FimR system.

*P. gingivalis* fimbriae expression is regulated by the microenvironment below the gumline and controlled by a variety of endogenous and exogenous factors. The biogenesis of fimA fimbria is controlled by the activation of the FimS-FimR two-component signal transduction system. FimR does not bind directly to the *fimA* promoter, but rather binds to the promoter region of the first gene (PG2130) in the *fimA* cluster. PG2130, in turn, regulates the expression of other genes in the *fimA* cluster, including PG2131 and PG2132, the *fimA* gene. In addition, using microarray experiments, it was reported that *fimR* mutant strains showed significant reduction in the expression levels of PG2133 and PG2134, postulating that FimA regulates the expression of PG2133 and PG2134 through an as yet unknown mechanism (Figure 1) [96]. Unlike *fimA*, *mfa1* gene regulation is accomplished by FimR directly binding to the promoter region of *mfa1* [97]. Previous studies showed that low temperature (34 °C) is necessary for the transcription of the *fimA* gene [98,99]. Additionally, the presence of the gingipains (RgpA and Kgp) is essential for the transcription of *fimA*, where *fimA* mRNA expression levels were significantly decreased in rgp and kgp mutant *P. gingivalis* strains [100]. Furthermore, the interaction of *P. gingivalis* with other periodontal pathogens influences the expression level of its fimbriae. It was shown that the development of *P. gingivalis* communities with *Streptococcus gordonii*, *Streptococcus sanguinis, and Streptococcus mitis* leads to downregulation of its minor fimbria [101], whereas the development of *P. gingivalis* communities with *Streptococcus cristatus* leads to the downregulation of the major fimbria [102].

### 1.4. Dendritic Cells: Revisiting the Dogma of DC Subsets and Differentiation

Dendritic cells (DCs) are antigen-capturing and -presenting cells (APCs) that play an important role in the innate immune system by capturing antigens and in the adaptive immune response by presenting the antigens to T-cells [103]. DCs have the ability to infiltrate several mucosal sites, including oral mucosa. Previous studies reported that DCs actively mobilize in and out of oral mucosal sites at different stages of periodontal health and disease [104,105,106,107], distinctly increasing in the lamina propria of PD tissues. DCs infiltrate the peripheral tissues in the immature state, where immature DCs capture and/or respond to a wide variety of microbes in the peripheral tissues via a set of extracellular as well as intracellular pattern recognition receptors (PRRs). PRRs identify pathogens via recognition of antigens such as pathogen-associated molecular patterns (PAMPs), including lipopolysaccharide (LPS), fimbriae, or flagellin [108]. Upon encountering a pathogen, PRRs activate a large number of complex intracellular signaling pathways, resulting in activation of gene expression and production of pro- and anti-inflammatory cytokines, chemokines, cell adhesion molecules, and immunoreceptors, orchestrating the early host response to invading pathogens and activation of the adaptive immune response [108]. Upon pathogen recognition and capture, immature DCs undergo maturation and must process the antigens and activate CD4+ T-cells and CD8+ T-cells via presenting antigens on their surface MHC-I and MHC-II molecules, respectively. The DC maturation process involves upregulation of co-stimulatory molecules (CD80, CD86), maturation markers (CD83), and antigen-presenting molecules (MHC classes I and II). Mature DCs then acquire a highly migratory profile through upregulation of chemokine receptors (e.g., CCR7) and secretion of cytokines (e.g., IL-12p70) [109]. Under optimum conditions, DCs migrate to secondary lymphoid organs and present the captured antigens to T-cells and prime naïve T-cells. As DCs migrate to lymphoid organs, blood DCs and monocytes migrate into the tissues and differentiate into DCs to replace migrating DCs and maintain DCs’ proper homeostasis, a cycle that is highly regulated by a variety of cytokines. 

DCs develop in the bone marrow from hematopoietic progenitors, expressing the transcription factor IRF8 [110] and the cytokine receptor FLT3 [111], which complete their differentiation in the periphery. DCs have evolved into multiple subsets throughout the body [112]. Conventional DCs (cDCs) are potent APCs and are classified into two major subsets. The cDC1 subset is specialized in presenting cell-associated antigens through cross presentation to CD8+ T-cells in addition to activating a type I immune response. On the other hand, the cDC2 subset (also called myeloid DC (mDC)), the focus of most of our work, is known to be more involved in activating CD4+ T-cells’ responses via MHC-II. Contrary to cDCs, the plasmacytoid DCs’ (pDCs) subset consists of relatively poor APCs and, instead, are more specialized in the rapid production of type I interferons (IFN-I); hence, they play an important role in responding to viral infection. In humans, DCs are generally divided into two major subpopulations, the pDC (CD11c^−^CD123^+^) and mDC (CD11c^+^CD123^−^) lineages. The mDCs can be further subdivided into CD141 (BDCA-3)^+^, CD16^+^ DC, and CD1c (BDCA-1)^+^ DC subsets [113,114,115]. The mDCs are further subdivided into CD141 (BDCA-3)^+^, CD16^+^ DC, and CD1c (BDCA-1)^+^ DC subsets [113,114,115]. In addition, DCs are subdivided into Langerhans cells and monocyte-derived DCs (MO-DC). Langerhans cells are present in the epidermis and oral mucosa and have a role in both tolerance and immune priming in that compartment. Mo-DCs differentiate from monocytes recruited in the event of tissue inflammatory responses and, in turn, instruct the differentiation of CD4^+^ T-cells into Th1, Th2, or Th17 cells [116,117,118].

### 1.5. Modulation of DC–T Cell Interaction by P. gingivalis

**Figure 2:**
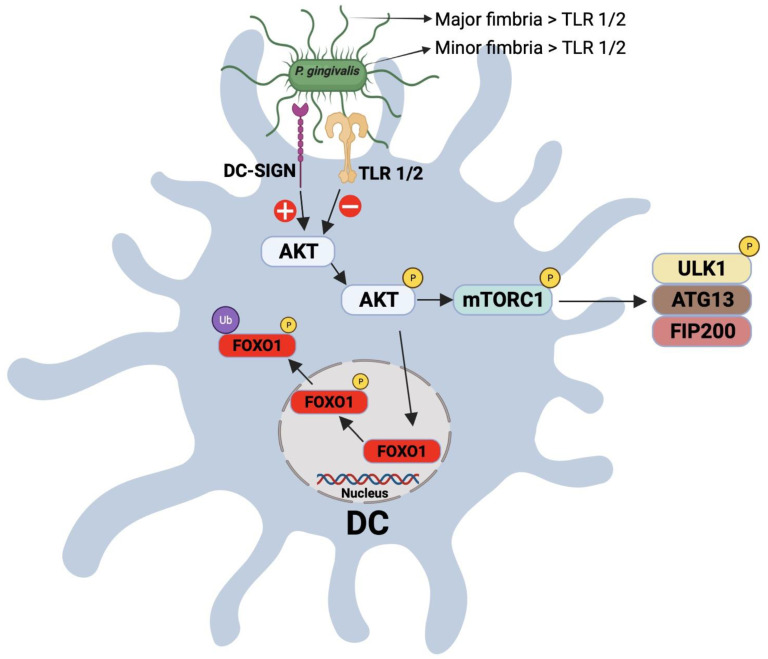
Schematic representation for the interaction between *P. gingivalis* fimbria and DC-SIGN and TLR receptors on DCs. Targeting of the DC-SIGN receptor on DCs results in the activation/phosphrylation of AKT, which, in turn, posphorylates and inactivates FOXO1, leading to the inhibition of apoptosis. Phophorylated AKT also phosphorylates mTORC1, resulting in its activation and inhibition of autophagy.

CD1c^+^(BDCA-1) CD209^+^ blood myeloid DCs have been shown to be expanded in subjects with PD, relative to healthy controls. More interestingly, it was also shown that this expansion further increases 24 h after mechanical debridement, which has been attributed to bacteremia [81]. In addition, myeloid DCs have been reported to be increased in PD patients with existing coronary artery disease. The increase in DC populations in the systemic circulation of a PD subject with coronary artery disease is associated with microbial carriage state of the DCs, most notably *P. gingivalis*. Postmortem analysis of coronary artery samples of coronary artery-diseased patients with PD shows co-localization of myeloid DCs’ marker, CD209 (DC-SIGN), with *P. gingivalis* minor fimbria protein (mfa-1) in the atherosclerotic plaques. Epidemiologic studies have reported that PD is associated with cardiovascular diseases, but the mechanism remains unclear. Recent studies implicated DCs in the microbial dissemination of periodontal pathogens. It was hypothesized that this was due to the manipulation of intracellular signaling in DCs by *P. gingivalis* minor fimbria via targeting the C-type lectin receptor DC-SIGN. Recently, it was shown that targeting DC-SIGN on DCs by *P. gingivalis* minor fimbria extends the survival of *P. gingivalis*-loaded DCs through the inhibition of apoptosis and autophagy [119]. Autophagy is a process whereby the cell disposes its intracellular damaged proteins and organelles by sequestering and directing cargo to the lysosome for degradation. Autophagy is crucial for the cell not only to maintain proper cellular homeostasis but also to defend against invading pathogens [120,121]. By trafficking intracellular bacteria to lysosomes, autophagy comprises an important element of the first line of defense, the innate immune response. Autophagy is involved in many immune functions such as clearance of intracellular pathogens [122,123,124], secretion of inflammatory cytokines [125], antigen presentation [126,127], and development of lymphocytes [128]. In addition, autophagy is regulated by a variety of immunological signals in response to the exposure of PRRs, such as TLRs and NLRs, to ligands or to cytokines. Moreover, TLR ligand-coated particles stimulate phagocytes and LC3-PE conjugation, a process called LC3-associated phagocytosis (LAP) [129]. Several in vitro studies reported the influence of *P. gingivalis* fimbria on autophagy in DCs. *P. gingivalis* has evolved an immune escape tactic whereby it evades intracellular killing in DCs by targeting DC-SIGN with its minor fimbria [122]. The same study reported that the intracellular killing of *P. gingivalis* inside DCs decreases while intracellular content increases via a DC-SIGN-dependent uptake of *P. gingivalis* by DCs. Furthermore, by blocking DC-SIGN by HIV glycoprotein 120, *P. gingivalis* survival inside DCs is reduced, but the mechanism of this phenomenon was unclear [122]. A more recent study showed that inhibition of autophagy in DCs by *P. gingivalis* involves targeting a crucial regulator of autophagy, the Akt-mTOR pathway [119]. *P. gingivalis* infection increases the expression of important elements in mTOR-dependent autophagy inhibition such as p-Akt Ser473, p-mTOR Ser2448, p-Raptor Ser792, and p-ULK1 Ser757 [119]. Other studies indicated that the hyperactivation of AKT [130] or of mTOR [131,132,133] plays influential roles in cellular senescence, with the mTOR inhibitor rapamycin obviating alveolar bone loss in mice [131]. Our work established a significant role for immune senescence induction by *P. gingivalis* in disabling the immune functions of DCs, including the ability of DCs to mature and induce antigen-specific T-cell proliferation [2]. Not yet clear is the role of major and minor fimbriae in promoting immune senescence (Figure 2). 

### 1.6. Significance of DC–T Cells’ Clusters in Periodontitis: Oral Lymphoid Foci

**Figure 3:**
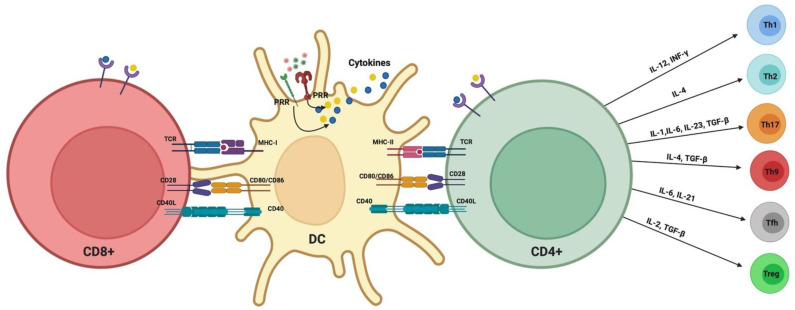
Classical DC–T-cell activation signals and polarization of T-cells.

When DCs were discovered in the early 1970s [134,135], they were thought to be predominantly immune stimulatory. It was decades later, in the 1990s, that the immune-regulatory functions of certain DC subtypes were recognized, including tolerogenic DCs [136,137]. Immature DCs monitor the periphery for antigens, which they phagocytose and process for antigen presentation to T-cells in the context of MHC-II molecules. DCs exposed to pro-inflammatory signals during antigen acquisition undergo maturation by upregulating their expression of MHC-II, co-stimulatory (e.g., CD40, CD80, CD86, CD83) lymph node-homing migratory chemokine receptors, and of inflammatory cytokines such as IL-12, while downregulating phagocytic mediators such as C-type lectins. DCs then migrate to regional lymph nodes, where they present their processed antigen peptides to T-cells in an immunostimulatory context, activating effector T-cell-type (e.g., Th1, Th17) responses [138]. We have reported in human studies the infiltration of oral lamina propria in PD with CD83+ matured DCs [139]. These mature DCs form immune complexes in situ with CD4+ T-cells [139]. These clusters in PD, called “oral lymphoid foci” [107], are analogous to ectopic lymphoid follicles found in many chronic inflammatory diseases [140] and are thought to result from continuous exposure to oral microbes and repeated damage to the oral mucosal epithelium [104,141,142]. In the experimental PD model in mice, a destructive role for in situ, matured DCs in promoting Th17-mediated alveolar bone loss was documented [143]. The exposure of DCs to innocuous antigens (e.g., apoptotic cells) in the absence of proinflammatory stimulants maintains their immature profile. In this scenario, very low levels of MHC-II-bound antigen peptides, co-stimulatory molecules, and secreted cytokines are expressed by DCs, inducing T-cell anergy [144]. TGF-β1 and IL-10 inhibit DC maturation and promote regulatory T-cell (Tregs) responses, while inhibiting Th17 effectors. This combination of cytokines loaded into DC-derived exosomes was shown to inhibit experimental PD in mice (Figure 3) [143].

## 2. Conclusions

Dendritic cells play a very active role in clearing infecting microbes, and other antigens when immature, and have been observed infiltrating oral mucosa at all stages of health and disease. Matured DCs are a unique feature of the established periodontitis lesion, forming immune conjugates with T-cells that are evocative of lymphoid tissues. These matured DCs promote Th17-mediated alveolar bone degeneration. The ability of *P. gingivalis* to coordinately regulate its fimbrial types, promoting or disabling DC maturation and Th17-type responses, should have a profound effect on the promotion or resolution of alveolar bone loss, most notably involving the process of immune senescence, though this will require further cause-and-effect studies in mice.
